# Analysis of risk factors and nursing care for nonspecific abdominal pain after endoscopic retrograde cholangiopancreatography (ERCP): A case report

**DOI:** 10.1097/MD.0000000000042637

**Published:** 2025-08-01

**Authors:** Ning Chen, Ben Wang, Jing Zhong, Kun Zhang

**Affiliations:** a Department of Digestive Endoscopy Center, Shandong Provincial Hospital Affiliated to Shandong First Medical University, Jinan, China; b Department of Gastroenterology, Shandong Provincial Hospital Affiliated to Shandong First Medical University, Jinan, China; c Department of Gastroenterology, Longkou People’s Hospital, Yantai, China; d Department of Pediatric Cardiology, Shandong Provincial Hospital Affiliated to Shandong First Medical University, Jinan, China.

**Keywords:** common bile duct stones, duodenal ulcer, ERCP, nasobiliary drainage, nonspecific abdominal pain, nursing care

## Abstract

**Rationale:**

Endoscopic retrograde cholangiopancreatography (ERCP) with endoscopic nasobiliary drainage is the standard treatment for common bile duct (CBD) stones. Post-ERCP abdominal pain is typically linked to complications like pancreatitis, perforation, or cholangitis. However, cases of nonspecific abdominal pain caused by duodenal ulcers induced by the nasobiliary drainage tube after ERCP have not been reported to date, highlighting the novelty and clinical relevance of this case.

**Patient concerns:**

A 48-year-old male with a history of recurrent upper abdominal pain presented with persistent upper abdominal tenderness following ERCP for CBD stones. Despite normal blood amylase and lipase levels, the patient experienced ongoing discomfort, prompting further investigation.

**Diagnoses:**

Preprocedure magnetic resonance cholangiopancreatography and upper abdominal magnetic resonance imaging confirmed stones in the lower CBD. Post-ERCP, follow-up gastroscopy revealed an ulcer in the duodenal descending segment, caused by incomplete displacement of the nasobiliary drainage tube.

**Interventions:**

The patient underwent ERCP to clear the CBD stones, with placement of a nasobiliary drainage tube. After identifying the duodenal ulcer secondary to tube displacement, the nasobiliary drain was removed, and targeted treatment and care were administered.

**Outcomes:**

Following removal of the displaced tube and appropriate management, the patient recovered rapidly and was discharged without further complications.

**Lessons:**

This case emphasizes the need for careful placement and monitoring of nasobiliary drainage tubes during ERCP to prevent displacement-related complications like duodenal ulcers. For patients with persistent nonspecific post-ERCP abdominal pain, gastroscopy is critical for differential diagnosis, as severe ulcers may lead to perforation. Clinicians and nurses should enhance their understanding of proper tube positioning and postprocedure assessment to improve patient outcomes.

## 1. Introduction

Common bile duct (CBD) stones are a prevalent clinical condition in which the stones obstruct the bile ducts, leading to biliary tract infections and secondary cholangitis. Typical symptoms include abdominal pain, chills, fever, jaundice (Charcot’s triad), and, in severe cases, hypotension and neuropsychiatric symptoms (Reynolds’ pentad). Endoscopic retrograde cholangiopancreatography (ERCP) is the primary treatment for CBD stones because of its minimally invasive nature, quick recovery, and cost-effectiveness.^[1]^ Post-ERCP complications may include acute pancreatitis, cholangitis, and perforation, which can affect rapid postoperative recovery. Abdominal pain after ERCP may indicate complications such as pancreatitis, perforation, or cholangitis^[1–3]^ but may also be due to nonspecific causes such as bloating or gastrointestinal spasms.^[2]^ Therefore, specialized intraoperative and postoperative care of ERCP patients is essential to prevent complications and nonspecific abdominal pain. This report describes a case from our hospital that analyzed the risk factors and precise treatment and care for nonspecific abdominal pain following ERCP for CBD stones.

## 2. Case analysis

**Figure 1.**
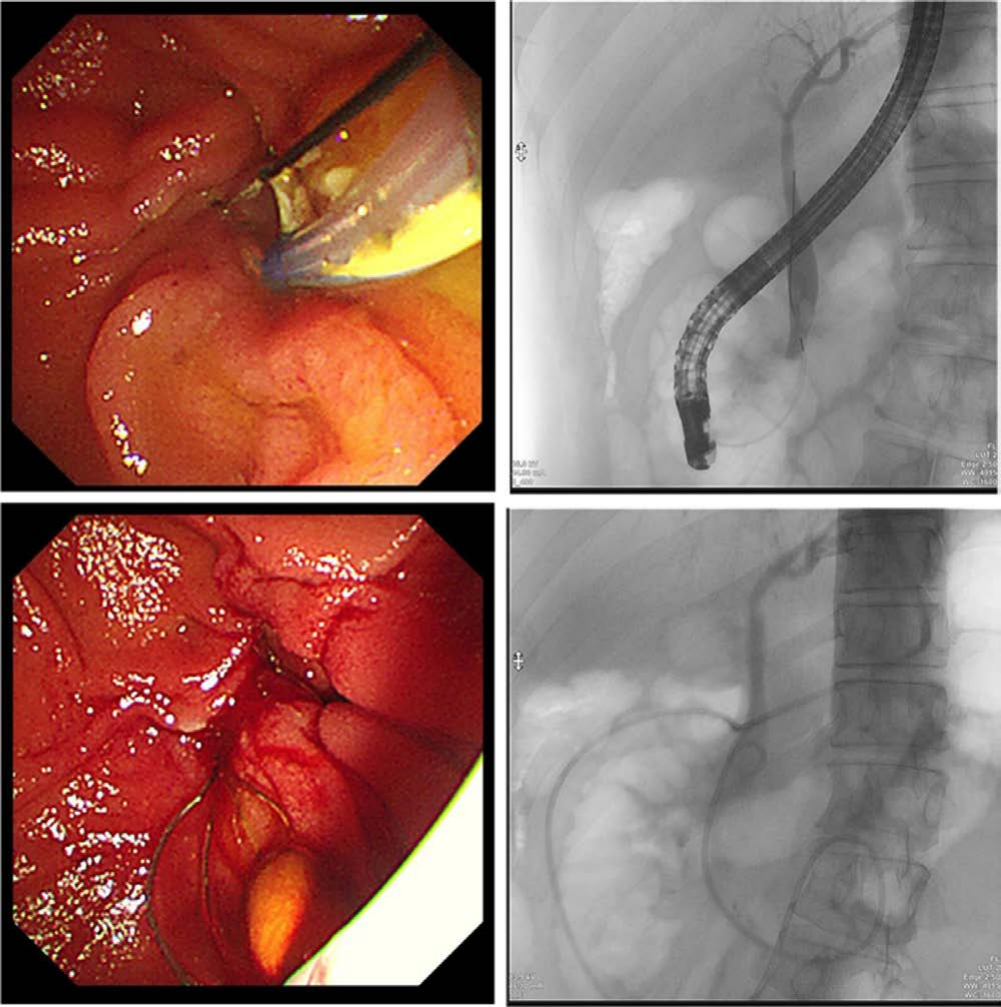
ERCP + EST + X-ray CBD stone + basket stone retrieval + balloon-sweeping ENBD. CBD = common bile duct, ENBD = endoscopic nasobiliary drainage, ERCP = endoscopic retrograde cholangiopancreatography, EST = endoscopic sphincterotomy.

A 48-year-old male was admitted to our hospital on March 4, 2024, with intermittent abdominal pain for over a year. The pain, which occurred after consuming fatty foods, resolved spontaneously without acid reflux, nausea, vomiting, belching, fever, headache, or cough. He had a history of elevated blood glucose levels for over a year, was managed with metformin, and was allergic to apples and peach skin. Magnetic resonance cholangiopancreatography and enhanced MRI of the upper abdomen revealed the following: Cholecystitis, stones in the distal CBD with intrahepatic and extrahepatic bile duct dilation, and duodenal diverticulum. Diagnoses included: CBD stones with cholangitis, and Cholecystitis, biliary duct dilation, duodenal diverticulum. On March 6, the patient underwent ERCP with endoscopic sphincterotomy, balloon dilation of the papilla, basket retrieval of stones, balloon sweep of stones, and nasociliary drainage, with successful stone removal (Fig. 1). The surgeon, an Associate Chief Physician (equivalent to a senior consultant), performed over 1000 ERCP procedures. The endoscopic equipment included a TJF-260V Olympus duodenoscope and CV-290 processor unit.

## 2.1. Procedure

The duodenoscope was then advanced into the descending duodenum. A triple-lumen sphincterotome with a guidewire successfully cannulated the CBD. Aspiration yielded yellowish bile, and cholangiography revealed a dilated CBD (12 mm) containing an 8 mm × 6 mm stone. Small endoscopic sphincterotomy followed by creeping balloon dilation (8 mm) was performed. The stone was extracted using a retrieval basket and clearance was confirmed using balloon occlusion cholangiography. Under fluoroscopy, a nasociliary tube was placed in the distal CBD and transnasally secured.

## 2.2. Postoperative course

POD (Postoperative day) 0: Stable with no symptoms.

POD 1: Moderate epigastric pain (visual analog scale 5/10) occurred after a liquid diet. Physical examination revealed epigastric tenderness, without rebound tenderness. The neutrophil count was 77.4% (normal: 40–75%). Laboratory tests (CBC, amylase, and lipase) were unremarkable.

POD 2: The bile culture grew *Escherichia coli*.

POD 3: Cefoperazone-sulbactam was initiated, and repeat labs remained normal.

**Figure 2.**
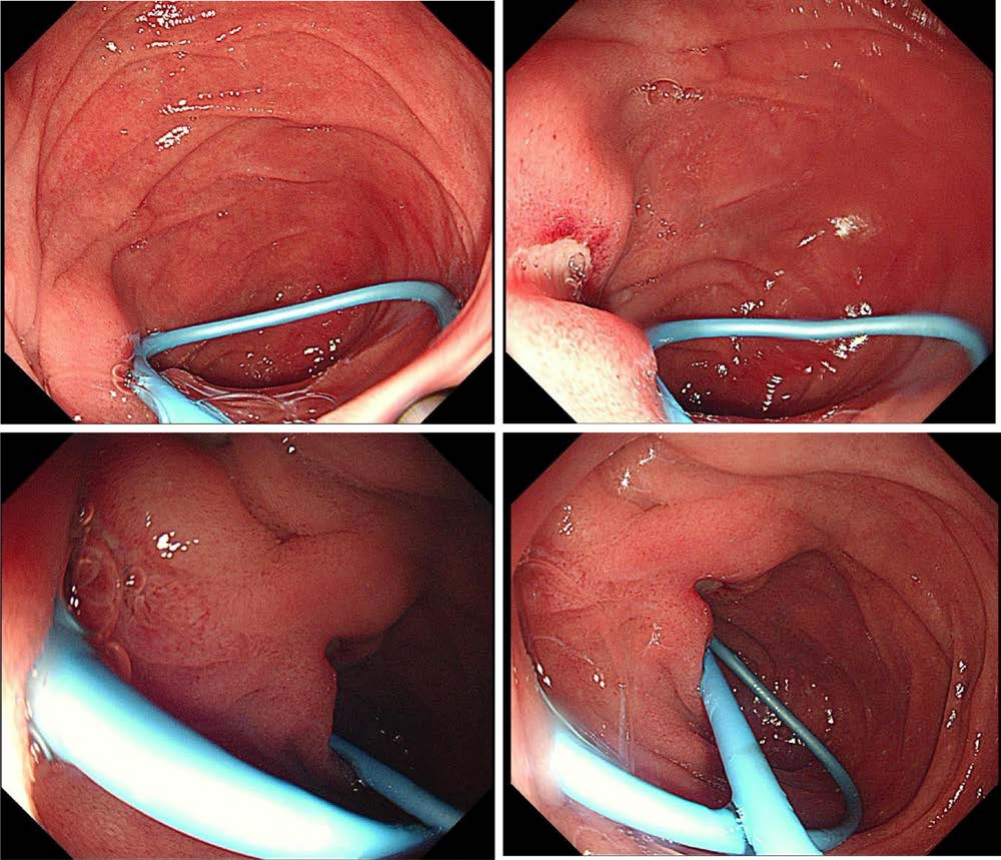
Gastroscopy showing a duodenal descending segment ulcer.

POD 4: Excluding complications such as pancreatitis, cholangitis, and perforation after ERCP, further gastroscopy examination of the duodenum was performed, gastroscopy revealed a fibrin-coated ulcer adjacent to the nasociliary tube in the descending duodenum (Fig. 2). The tube was removed and pain was reduced to a visual analog scale score of 3/10.

POD 7: Complete resolution of symptoms (refer to Table 1).

**Table 1:** Medical record timeline

Time	Event
March 4, 2024	A 48-year-old male was admitted to the hospital. He had intermittent abdominal pain for over a year, which occurred after consuming fatty foods and resolved spontaneously. There were no acid reflux, nausea, vomiting, belching, fever, headache, or cough. He had a history of elevated blood glucose for over a year, treated with metformin, and was allergic to apples and peach skin. MRCP and enhanced MRI of the upper abdomen showed cholecystitis, stones in the distal common bile duct with intrahepatic and extrahepatic bile duct dilation, and duodenal diverticulum. Diagnoses were common bile duct stones with cholangitis, cholecystitis, biliary duct dilation, and duodenal diverticulum
March 6, 2024	The patient underwent ERCP with endoscopic sphincterotomy (EST), balloon dilation of the papilla, basket retrieval of stones, balloon sweep of stones, and nasobiliary drainage. The duodenoscope was advanced into the descending duodenum. A triple–lumen sphincterotome with a guidewire cannulated the common bile duct (CBD). Aspiration showed yellowish bile, and cholangiography revealed a 12-mm dilated CBD with an 8 mm × 6 mm stone. Small EST and 8-mm creeping balloon dilation were performed. The stone was extracted with a retrieval basket, and balloon occlusion cholangiography confirmed clearance. A nasobiliary tube was placed in the distal CBD and transnasally secured
POD 0	The patient was stable with no symptoms
POD 1	After a liquid diet, the patient experienced moderate epigastric pain (VAS 5/10). Physical examination showed epigastric tenderness without rebound tenderness. The neutrophil count was 77.4% (normal: 40–75%). CBC, amylase, and lipase tests were normal
POD 2	Bile culture grew *Escherichia coli*
POD 3	Cefoperazone–sulbactam treatment was started, and subsequent lab tests remained normal
POD 4	Excluding complications such as pancreatitis, cholangitis, and perforation after ERCP, further gastroscopy examination of the duodenum was performed, gastroscopy showed a fibrin–coated ulcer adjacent to the nasobiliary tube in the descending duodenum. The tube was removed, and the pain reduced to a VAS score of 3/10
POD 7	The patient’s symptoms completely resolved

Professional and personalized care was provided as follows.

## 3. Nursing issues

### 3.1. Causes of abdominal pain

Postoperative pancreatitis: Associated with surgical risks such as difficult cannulation, guidewire entry into the pancreatic duct, or pancreatic duct imaging and sphincterotomy.^[3]^

Perforation: Related to cannulation of the duodenal papilla, sphincterotomy, balloon dilation, and duodenal diverticulum.^[1,3]^

Cholangitis: Associated with basket retrieval of stones and balloon sweeps.^[1]^

Nonspecific pain: Related to abdominal bloating, gastrointestinal spasm, or friction between the nasociliary tube and duodenum.^[2]^

### 3.2. Nursing goals

Identify risk factors for abdominal pain, provide precise intraoperative coordination, and provide personalized postoperative care to alleviate abdominal pain.

### 3.3. Nursing measures

Preoperative care: Routine rectal indomethacin suppositories, if not contraindicated.^[3]^

Intraoperative care: Avoid unnecessary pancreatic duct imaging and placement of a pancreatic duct stent in high-risk patients with acute pancreatitis.

Risk factors for perforation: For elderly female patients, those with gastrointestinal reconstruction, diverticula or ulcers, or difficult stone retrieval should be vigilant during sphincterotomy and balloon dilation. CO_2_ is used instead of air to prevent accidental perforation.^[1]^

Nasobiliary tube placement: The small curved end of the nasociliary tube was placed in the upper segment of the CBD to prevent incomplete removal, leading to duodenal ulcers. Avoid friction-induced ulcers and perforations.

Monitoring and observation: Vital signs, patient consciousness, and temperature. Perform electrocardiogram monitoring if necessary. Abdominal pain, bloating, fever, and changes in the abdominal signs were observed. Blood amylase and white blood cell (WBC) counts were regularly measured and recorded to detect abnormalities early and to notify the physician.

Dietary care: Fast for 24 hours postoperatively to avoid stimulating pancreatic secretions. Wet cotton swabs or warm water was used to relieve dry mouth. After 24 hours, if amylase levels were elevated, participants continued fasting or consumed clear liquid foods. Resume a low-fat, semisolid diet once amylase levels normalize, with gradual progression to regular food.

Nasobiliary tube care: Ensure that the tip of the drainage tube is in the upper CBD, with a nasociliary tube in the duodenal papilla. Fix it externally and instruct the proper fixation techniques. The drainage bag was replaced daily and sterility was maintained. Regularly check for displacement, kinks, compression, or blockage. If significant white flakes or sediment were observed in the drainage fluid, bile duct flushing with gentamicin and saline was performed to prevent and control the infection, ensuring a strict sterile technique. The color, shape, and volume of drainage fluid were monitored and recorded. If the fluid is discolored or decreased, consider blockage and flush the tube, possibly using X-ray imaging for re-cannulation if necessary. Cloudy drainage suggests infection, whereas blood-stained fluid indicates bleeding, which requires prompt reporting and treatment.^[4]^ Postoperatively, the drainage fluid may contain a small amount of flocculent material for 1 to 2 days. It generally transitions to a brownish-yellow or light-yellow color within 3 to 4 days. The drainage volume decreased and the color decreased over time. The typical 24-hour drainage amount is 800 to 1000 mL.

Complication management: Monitoring for abdominal pain, signs of peritoneal irritation, and blood and urine amylase tests within 24 hours to exclude pancreatitis or bowel perforation. Reports anomalies promptly and provides treatments, such as fasting, gastrointestinal decompression, anti-inflammatory, antispasmodic, and pancreatic secretion suppression.^[5]^ Observe electrolyte imbalances and prevent disturbances in the acid-base balance.^[2,6]^

Psychological support: Use narrative dialogue to enhance patient understanding of postoperative abdominal pain and foster a positive attitude to accelerate recovery. Assess pain levels and employ relaxation techniques such as listening to music or deep breathing. Engage family members for emotional support and provide encouragement and comfort to patients.^[7,8]^

Early mobilization: Encourage out-of-bed activities and limb exercises without contraindications within 4 hours post-surgery, gradually increasing activity.

Narrative nursing: Listen to patients, conduct early identification of patients, and eliminate the risk factors of abdominal pain after ERCP. Provide health education and humanistic care, help patients relieve their tension and anxiety, enable them to accept gastroscopic examination and treatment, and promote their recovery.

## 4. Discussion and conclusion

Post-ERCP abdominal pain may indicate complications, such as pancreatitis, cholecystitis, perforation, or nonspecific causes.^[2]^ The characteristics of post-ERCP pain vary and the presence of abdominal or systemic signs may differ. The differential diagnosis of postoperative pain is as follows: Postoperative pancreatitis is characterized by new or worsened abdominal pain with amylase or lipase levels exceeding 3 times the normal value within 24 hours.^[3]^ Postoperative cholecystitis presents with signs of right upper abdominal inflammation (e.g., Murphy’s sign and tenderness), systemic inflammation (e.g., fever, elevated C-reactive protein level, and WBC count), and characteristic imaging findings, with no pre-ERCP indications of cholecystitis.^[9]^ Perforation is marked by immediate severe abdominal pain, right upper quadrant muscle rigidity, subcutaneous emphysema, respiratory distress, decreased oxygen saturation, elevated WBC or neutrophil counts, and possible fever with increased amylase and upper abdominal pain.^[1,10]^

Acute pancreatitis is the most common post-ERCP complication, occurring in 3.0% to 14.7% of cases with a mortality rate of 0.1% to 1.1%.^[11–13]^ Although extensively studied, research on nonspecific pain is limited. Some reports have suggested that nonspecific pain may be related to bowel dysfunction and typically resolves within a few hours.^[1,2,14]^ The early identification of nonspecific pain and its risk factors remains challenging.

In this case, normal amylase and lipase levels at 24 hours and day 4, elevated neutrophil percentages, and abdominal tenderness without rebound pain suggested nonspecific pain. On day 4, gastroscopy revealed an incomplete nasociliary tube causing a friction-induced duodenal ulcer, leading to its removal and pain relief. This case was finally confirmed to have nonspecific abdominal pain caused by a duodenal ulcer through a gastroscopy examination, and follow-up showed pain resolution after 3 days.

Through a search on PubMed, the literature report^[15]^ presents a case demonstrating the potential of endoscopic treatment for the perforation of refractory duodenal ulcers. By endoscopically indwelling a nasociliary drainage tube to drain bile, it can inhibit the activation of tissue kallikrein into kallikrein, prevent enterokinase from activating trypsinogen into trypsin, restrain the progression of tissue damage at the ulcer site, relieve peritonitis, improve renal function, and further enhance the overall condition of the patient, thus promoting the healing of duodenal ulcers. As can be seen from the literature reports,^[16,17]^ nasociliary drainage promotes the healing of duodenal ulcers, and endoscopic nasociliary drainage is an effective method for treating duodenal ulcers, refractory bleeding, and preventing perforation of duodenal ulcers. The literature report^[18]^ describes a case in which conservative treatment of a perforated duodenal diverticulum with an enterolith was successfully achieved by indwelling a nasociliary drainage tube within the diverticulum, avoiding the need for surgical operation, making it an effective approach for treating perforation of duodenal diverticula.

By reviewing this case, in which the nasociliary drainage tube is incompletely displaced to the end of the CBD during ERCP, it is recommended that during the ERCP procedure, a curved nasociliary drainage tube be placed along the guidewire under X-ray fluoroscopy to confirm its position in the middle and upper segments of the CBD. This is to avoid placing the tube at the end of the CBD, thereby preventing duodenal ulcers caused by the incomplete displacement of the nasociliary drainage tube. This approach aims to avoid placing the tube at the end of the CBD, thereby preventing duodenal ulcers caused by the incomplete displacement of the nasociliary drainage tube. For predictive nursing care after ERCP, it is crucial to identify risk factors at an early stage and implement corresponding nursing intervention measures promptly. This can help alleviate pain and promote rapid recovery of the patients.

This unique case highlights the importance of identifying and addressing specific causes of post-ERCP abdominal pain to improve patient management and outcomes.

In summary, precise treatment and professional, personalized care for nonspecific pain following ERCP for CBD stones resulted in rapid recovery. This case underscores the need for early identification of risk factors and interventions to enhance the quality of ERCP management.

## Acknowledgments

I am very grateful to all physicians, nursing staff, and other caregivers of the The Gastroenterology Division of Shandong Provincial Hospital is Affiliated with Shandong First Medical University.

## Author contributions

**Data curation:** Jing Zhong.

**Supervision:** Ben Wang.

**Writing – original draft:** Ning Chen.

**Writing – review & editing:** Kun Zhang, Ning Chen.
